# The Role of Resources on Job Satisfaction among US Public Health Master’s and Doctoral Program Graduates at the Intersection of Race, Ethnicity, and First-Generation Status

**DOI:** 10.1007/s10900-025-01513-2

**Published:** 2025-09-11

**Authors:** Kimberly Wu, Felicia Setiono, W. Marcus Lambert, Shokufeh Ramirez, Christine M. Arcari, Katherine P. Theall, Dovile Vilda

**Affiliations:** 1 Department of Social, Behavioral, and Population Sciences, Celia Scott Weatherhead School of Public Health and Tropical Medicine, New Orleans, LA 70112 USA; 2 SUNY Downstate Health Sciences University, Brooklyn, NY 11203 USA; 3 Department of Epidemiology , School of Public Health and Tropical Medicine , Celia Scott Weatherhead, New Orleans, LA 70112 USA

**Keywords:** Workforce readiness; public health, First generation students, Graduate students

## Abstract

Investing in a diverse public health workforce has implications for strengthening cultural humility and addressing health inequities within minoritized populations. First-generation (FG) students pursuing graduate level degrees are an important population with the potential to strengthen such efforts in their transition into the public health workforce. However, research on the factors influencing job satisfaction and job decisions is limited. The main objective of this study was to examine how personal, social, and economic resources influence job satisfaction among public health master’s and doctoral level graduates, and to explore differences across education generation and racial/ethnic background. Drawing on Conservation of Resource and Intersectionality theories, we conducted secondary analysis using a recent national survey investigating factors that are associated with public health career choices (*n* = 751). Adjusted and weighted linear regression models, both with and without interaction terms, were analyzed to examine the associations between resource domains and job satisfaction. Our findings revealed significant moderating effects of FG status across personal, social, and economic resource domains in shaping job satisfaction, both within the full sample and among specific racial and ethnic subgroups. Further analysis revealed stronger influence of these resources among Black FG, White FG and total FG groups compared to non-FG groups, suggesting these resources may play a role in influencing job satisfaction among first-generation individuals. Therefore, public health graduate level academic programs and employers should consider strategies that improve access to resources to better support first-generation individuals’ completion of degrees and transition into the workforce.

**Supplementary Information:**

The online version contains supplementary material available at 10.1007/s10900-025-01513-2.

## Introduction

A diverse public health workforce positively impacts public health infrastructure by enhancing cultural humility and building trust in health resources and research. By including minoritized populations and supporting diverse research priorities, public health and healthcare systems can more effectively meet the needs of minoritized populations in the U.S [1–4]. Much of the literature on strengthening the public health workforce focuses on the dual strategies of diversifying public health training programs and improving the recruitment and retention of workers from underrepresented populations [1, 5, 6]. Central to these efforts must include the recognition of the unique place of first-generation (FG) students, those who are the first among their families to pursue undergraduate degrees, as a critical population [7]. Prioritizing their inclusion and success is foundational to achieving an equitably diverse public health workforce.

Yet, limited research exists on the experiences of first-generation students navigating public health training to workforce pathways, as compared to studies focusing on first-generation students in medicine [8–10], dentistry [11, 12], nursing [13, 14], and pharmacy graduate programs [15, 16]. Furthermore, the literature on FG students often prioritizes undergraduate experiences, suggesting that this group often face additional challenges as compared to their peers from college educated families when navigating their higher education journeys [17–19]. Unique challenges faced by undergraduate FG students include their need for academic preparation, financial burdens, and lack of social support from their peers, many of which can persist into their experiences as graduate students and early career professionals [20–22].

There is less research on FG graduate students and their transition to the workforce but, so far, emerging literature has documented mental health challenges and stressors faced by FG graduate students [23–26]. In Germany, researchers found that compared to the general population, graduate students overall are six times as likely to experience depression. Furthermore, a systematic review of the existing literature on the topic showed that entering and adapting to graduate-level programs served as a common source of stress for all students, suggesting that these challenges may similarly affect FG individuals pursuing graduate level degrees [27, 28]. Building on these studies, emerging research highlights that FG students of color who go on to faculty and administrative positions faced burdens related to feelings of isolation and structural barriers on campus, which can intensify the stressors of adapting to their graduate training [29]. Ultimately, a lack of literature on this topic points to the need for more efforts to study the experiences of graduate students in public health to enhance our understanding of their unique challenges and outcomes.

In addition, limited evidence can be found on the experiences of FG graduate-level public health professionals in the workforce. Existing literature highlights disparities between FG and non-FG professional outcomes in the workforce, including lower rates of FG graduates than non-FG graduates employed in positions requiring a college degree, and lower annual salaries for FG professionals than non-FG professionals [30]. A joint 2019 report from the Office of Civil Rights and the U.S Census Bureau, highlighted workplace barriers faced by FG professionals linked to disparate access to “(1) developmental programs and internships before college, (2) educational and professional networks, (3) disposable income for social events with coworkers, (4) orientation on how to navigate office culture and advance one’s career, and (d) career mentors” [31] (pg. 2).

Lastly, the intersectional experiences of FG public health professionals of color remain underexplored. Understanding trends in career selection for this population and addressing structural factors, such as the impact of resources, can inform efforts to maintain a dynamic and diverse public health workforce with attention to enhancing employee retention. For instance, job satisfaction can play a critical role in workforce stability and employee well-being [32]. Findings from a meta-analysis of studies on general employee experience and health have shown job satisfaction to be associated with changes in mental health for burnout, depression, anxiety, and self-esteem [32]. Job satisfaction has also been recognized as an important factor for staff retention for governmental public health positions [33]. However, few studies on the topic have incorporated theoretical frameworks to explore the structural factors influencing job satisfaction.

Conservation of resource theory (COR) can enhance our understanding of how structural factors influence FG professionals’ job satisfaction through the mechanisms of managing resource loss, gain, and balance. For instance, Barboza-Wilkes and colleagues adopted COR theory in their analysis on the dimensions of burnout (depersonalization and loss of personal accomplishment) among government employees in California [34]. According to the COR theory, more focus needs to be placed on understanding how individuals prioritize and make decisions based on dynamics of resource loss and gain [35]. COR theory could enhance current literature by identifying other sources of structural and individual support and stress relevant to FG professionals in public health by investigating their role in factors influencing workplace experiences, such as job satisfaction. Furthermore, given the lack of intersectional research into the experiences of FG public health professionals, incorporating intersectionality with COR theory can highlight comparative differences in job satisfaction and work experience between and within racially and ethnically minoritized groups [36].

Going beyond its origins in the legal field, intersectionality allows researchers to explore the greater complexity of the interlocking effects of macro-level oppression influencing individuals’ lives [37]. To operationalize an intersectionality framework for the purposes of public health research, two main approaches to researching the relationship of categories are often highlighted: inter- and intra-categorical approaches [38]. While intercategorical research highlights comparisons between intersections of identity, intracategorical analysis allows for focus within intersections of identity [38]. In the context of this study, intersectionality enhances the investigation into the differences of needs and outcomes within the sample broadly and more specifically within certain education generation and racial/ethnic identities.

Drawing on the intersectionality and conservation of resource theory, this study was guided by three objectives and utilized secondary survey data of a sample of recent public health master’s and doctoral level graduates (referred to from here as graduates): (1) to identify the key public health employment sectors among graduates by diverse identities based on education generation, racial/ethnic backgrounds and gender, (2) to determine the relationship between personal, social, and economic resources on levels of job satisfaction among first-generation graduates of color (FGGOC) in the public health workforce, and (3) to investigate how identifying as first-generation moderates the relationships between personal, social, and economic resources and job satisfaction for recent public health graduates.

Accordingly, we hypothesized that first, there will be differences in current job sector of employment by education generation status, racial/ethnic backgrounds, and gender identities. Second, we anticipate that associations between personal, social, and economic resources and job satisfaction will differ between FGGOC and FG White graduates. Lastly, we hypothesized that the associations of personal, social, and economic resources on job satisfaction will vary depending on individuals’ intersecting identities (especially by FG or non-FG statuses).

## Materials and Methods

### Study Design and Participants

This cross-sectional study was part of a larger research initiative that explores the training and workforce experiences of students of color pursuing graduate education in public health at the State University of New York (SUNY) Downstate Health Sciences University. The present study utilized a subset of this data to examine the workforce experiences of recent graduates completing master’s and doctoral-level public health programs across the U.S., focusing on the experiences of first-generation graduates of color.

A national survey exploring factors that influence public health career choices was disseminated by SUNY Downstate from March 1, 2023, to June 1, 2024. The SUNY Downstate team employed purposive sampling to identify CEPH-accredited public health education institutions and professional associations for recruiting recent public health graduates. Recruitment included sharing the survey link and relevant information with alumni networks, public health professional associations, and via social media (LinkedIn, Twitter (now called X)). Efforts were made to connect with professional associations (i.e.: caucuses within American Public Health Association (APHA) and the national McNair Scholars Program, which supports FG students and underrepresented minorities in higher education). The anonymous survey was distributed to 165 CEPH-accredited programs and 66 national public health associations across the U.S. The current study conducted secondary analysis using the survey responses.

A total of 1,098 responses were collected. After data cleaning and quality assurance checks, the final sample was drawn from 751 responses who completed the full survey, had valid data for variables of interest, and completed their degrees within the last 20 years (2004–2020, mean: 2019). The study sample included representation from 125 universities from around the United States (43 private and 82 public universities). Despite efforts to oversample FG individuals, this group was still underrepresented compared to the national average of first-generation public health graduates from the 2021 National Science Foundation’s National Survey of College Graduates (28.4% vs. 35.3%). Data was collected through a self-administered survey via Qualtrics™, a web-based tool, capturing demographic information, public health training, recent job history, job satisfaction, and barriers and facilitators influencing workforce entry. A copy of the relevant survey questions for analysis is included in Supplemental Materials. The survey received IRB approval from SUNY Downstate’s School of Public Health (IRB Study #1929526).

### Measures

The parent survey collected data on factors influencing career decisions among public health graduates. It included demographic questions to gather background information about respondents’ identities and details about their most recent public health graduate training (i.e., institution, year of graduation, type of degree conferred). In addition, the survey asked about respondents’ first job upon entering the public health workforce, the employment sector of that first job, their current job in public health, and the current employment sector. A series of questions explored reasons behind respondents’ decision to find a job in public health, as well as the factors influencing their decision to work in their current employment sector.

### Outcome

The outcome of interest was the self-reported level of satisfaction with their current job. Job satisfaction was measured using a composite score derived from six indicator items scored on a 5-point Likert scale, where “Strongly Agree” = 1 to “Strongly Disagree” = 5 (Question 15, Appendix 2). The six indicator items for job satisfaction were as follows: (1) “I am happy with my current employment sector”, (2) “I am happy with the amount of opportunities to advance in my current position”, (3) “I am happy with my current salary”, (4) “I am happy with the level of responsibility in my position”, (5) “I enjoy the people that I work with”, and (6) **“**I hope to stay in my current employment sector long term”. The items were recoded so that higher scores reflected greater job satisfaction. The composite measure (ranging from 6 to 30) demonstrated strong internal consistency reliability, with a Cronbach alpha of 0.82.

### Exposure

The primary exposure variables included measures of personal resources (self-efficacy), social resources, and economic resources. Survey items were categorized post hoc to construct composite measures for each resource domain guided by the Conservation of Resource (COR) theory [39].

### Personal Resources Domain

Personal resources were operationalized as self-efficacy (Question 19, Appendix 2), measured using the New General Self-Efficacy Scale [40], a validated measure comprised of eight items that assess an individual’s belief in their ability to achieve an outcome while overcoming difficulties. Each item was scored on a 5-point Likert scale with 1= “Strongly Disagree” to 5= “Strongly Agree”, with higher scores corresponding to higher levels of self-efficacy [40]. Example items include statements such as “I will be able to achieve most of the goals that I set for myself”, “When facing difficult tasks, I am certain that I will accomplish them”, “I am confident that I can perform effectively on many different tasks”, and “Even when things are tough, I can perform quite well” [40] . The eight items were combined into a composite measure that demonstrated strong internal consistency reliability (Cronbach’s alpha = 0.90). Higher levels of self-efficacy scores corresponded with greater levels of personal resources (range 8–40), with the sample range from 15 to 40.

### Social Resources Domain

Social resources were also operationalized as a composite score measured using eleven indicator items from Questions 16 and 17 (Appendix 2). Example items include: (1) “Guidance from mentors has highly influenced my current employment sector”, (2) “The choice of my current employment sector was highly influenced by formal career counseling in my graduate education”, (3) “The choice of my current employment sector was highly influenced by my family’s occupational background (e.g., I have a family member who is in academia/government/a healthcare organization or hospital, etc.)”, and (4) “The choice of my current employment sector was highly influenced by responsibility to my family (significant other/spouse, children, and/or other dependents)”. The indicator variables were measured on a 5-point Likert scale, from “Strongly Agree” =1 to “Strongly Disagree” = 5. The items were recoded so that higher scores reflected higher levels of social resources influencing job decisions. The composite measure (11–55) had a sample range from 13 to 55, demonstrating acceptable internal reliability, with a Cronbach’s alpha of 0.75.

### Economic Resources Domain

Economic resources were operationalized with a composite of the following three items from Question 16: (1) The choice of my current employment sector was highly influenced by my level of student debt, (2) The choice of my current employment sector was highly influenced by my need (or desire) to earn more money, (3) The choice of my current employment sector was highly influenced by the financial prospects of the career sector. The indicator variables were scored using the 5-point Likert scale, with “Strongly Agree” =1 to “Strongly Disagree” = 5. Items were recoded so that higher scores reflected greater value of financial needs when making job decisions. The composite measure (ranging from 3 to 15) demonstrated moderate but below-threshold internal consistency, with a Cronbach’s alpha of 0.67.

### Covariates and Moderating Variables

Several covariates were included in this study. Gender was measured as a binary variable to account for males and females. Age was parsed into 5 interval categories, with those ages 24 or younger serving as the reference group, including groups for those 25–34, 35–44, 45–54, and 55 and above. Financial assistance to fund graduate public health degrees was measured as a binary variable to identify those who received some form of financial aid (1) and those who did not (0). Degree type was included as a binary variable to distinguish between master’s-level and doctoral-level public health degrees. Master’s-level degrees included the MPH and Master of Science (MS), while doctoral-level degrees included the DrPH and Doctor of Philosophy (PhD) in Public Health. Marital status was included as a binary variable, distinguishing between individuals who were married (1) and those who were not married, including divorced, separated, or never married (0). Key moderating variables included education generation status and self-reported racial/ethnic identities. Education generation status was included as a binary variable where 1 = first generation and 0 = continuing generation (non-FG). Racial and ethnic subgroups included Asian, Black, Hispanic, Other, and White. The Other subgroup was created due to the small sample of respondents identifying as Native American/American Indian, Pacific Islander/Native Hawaiian, Middle Eastern & North African, and multiracial.

### Data Analysis

Descriptive and bivariate statistics were used to summarize the sample based on levels of job satisfaction and job sectors of recent employment by education generation status, racial/ethnic background, and gender. Measures for resources (personal, social, and economic) and other covariates, such as degree type and type of institution where participants earned graduate-level public health degrees were also examined. The data was stratified by race and education generation status to explore experiences across 10 intersectional identities by education generation status and race (e.g., FG Asian, FG Black, non-FG White).

Bivariate analysis was conducted to examine crude associations between economic, social, and personal resources and job satisfaction, both overall and stratified by race and education generation status. Given the small sample sizes in stratified analyses, Mann-Whitney U tests were used to compare ranked means of job satisfaction scores, as well as measures for personal, social, and economic across education generation and racial/ethnic background. This type of analysis falls within descriptive inter-categorical intersectionality [41].

Adjusted linear regression models, stratified by education generation status and by race, were constructed for each exposure and their associations with job satisfaction was examined. According to Bauer and Schiem, this type of analysis can be described as analytic intersectionality (Bauer & Scheim, 2019). Final models were developed using a directed acyclic graph (DAG) approach to identify relevant covariates, account for interaction effects, and to apply weighting. Weights for the model were created using the 2021 National Science Foundation’s National Survey of College Graduates data [42]. Proportions based on gender, racial and ethnic identity, and first-generation status were calculated, and the final weight was constructed using these proportions.

To assess the interaction effect of intersecting identities on the relationships between resources and job satisfaction, education generation status (FG or non-FG) was interacted with each resource domain within each racial subgroup. In models where interaction effects were not significant (i.e., *p* > 0.20) [43], the interaction term was removed from the final models for parsimony and interpretability. To assess the extent of significant interaction effects, models were created by further stratifying for race/ethnicity and education generation status group. In these models, the Other subgroup was dropped from analysis given its small sample size. All analyses were conducted in RStudio (Version 2023.12.1 + 402).

## Results

### Participant Characteristics

Table 1 presents the sample characteristics, stratified by education generation status (*n* = 751). First-generation individuals made up 28.4% of the sample, whereas non-FG individuals made up 71.6% of the total sample. Most of the study sample self-identified as White (51.8%), female (82.2%), between the ages of 25–34 (61.8%), U.S. citizens (92.3%), never married (55.9%), not receiving financial assistance for graduate school (65%), attending a private institution for their graduate degrees (54.7%), and completing a master’s-level public health degree (90.1%).

**Table 1:** Characteristics of study participants (Unweighted)

	First-Generation (*n* = 213)		Non-First Generation (*n* = 538)		Chi-Square Results	Total (*n* = 751)	
	n	%	n	%	p-value	n	%
*Race/Ethnicity*							
Asian	33	15.5%	80	14.9%	0.733	113	15.0%
Black	38	17.8%	64	11.9%	**0.003**	102	13.6%
Hispanic	43	20.2%	37	6.9%	**< 0.001**	80	10.7%
Other	18	8.5%	43	8.0%	0.967	61	8.1%
White	80	37.6%	309	57.4%	**< 0.001**	389	51.8%
*Gender*							
Male	47	22.1%	70	13.0%	**0.003**	117	15.6%
Female	162	76.1%	455	84.6%	**0.003**	617	82.2%
Non-binary	4	1.9%	13	2.4%	0.790*	17	2.3%
*Age*							
24 year or younger	11	5.2%	32	5.9%	0.804	43	5.7%
25 to 34	127	59.6%	337	62.6%	0.476	464	61.8%
35 to 44	46	21.6%	123	22.9%	0.772	169	22.5%
45 to 54	18	8.5%	31	5.8%	0.240	49	6.5%
55 and above	11	5.2%	14	2.6%	0.111*	25	3.3%
*Low Income Loan eligibility*							
Yes	124	58.2%	118	21.9%	**< 0.001**	242	32.2%
No	84	39.4%	404	75.1%	**< 0.001**	488	65.0%
Unsure	5	2.3%	16	3.0%	0.808*	21	2.8%
*Institution Type*							
Public	117	54.9%	192	35.7%	**< 0.001**	309	41.1%
Private	85	39.9%	326	60.6%	**< 0.001**	411	54.7%
International	8	3.8%	16	3.0%	0.645*	24	3.2%
*Degree Type*							
Master’s	186	87.3%	491	91.3%	0.115	677	90.1%
Doctorate	27	12.7%	46	8.6%	0.115	73	9.7%
*Marital Status*							
Married	83	39.0%	217	40.3%	0.750	300	39.9%
Never married	121	56.8%	299	55.6%	0.883	420	55.9%
Other (widowed, divorced, separated)	9	4.2%	19	3.5%	0.672*	28	3.7%
*US Citizenship*							
Yes	198	93.0%	495	92.0%	0.896	693	92.3%
No	15	7.0%	39	7.2%	1.000	54	7.2%
Choose not to answer	-	-	2	0.4%	-	2	0.3%

*Computed with Fisher’s exact Chi-Square test

We observed significant differences between FG and non-FG participants in several areas, including race/ethnicity (Black, Hispanic, and White representation), gender (male vs. female), eligibility for low-income loans, and the type of institution attended for a graduate-level public health degree. For instance, a significantly higher percentage of FG participants identified as Black (17.8%), Hispanic (20.2%), and male (22.1%) as compared to non-FG participants (11.9%, 6.9%, and 13% respectively). Furthermore, among FG individuals, more than half (58.2%) reported receiving some form of financial assistance for their public health graduate training, in contrast to over 75% of non-FG individuals who reported not receiving financial assistance. Additionally, over half (54.9%) of FG individuals attended a public institution for their graduate studies, whereas a majority of non-FG individuals predominantly attended private institutions (60.6%).

**Fig. 1:**
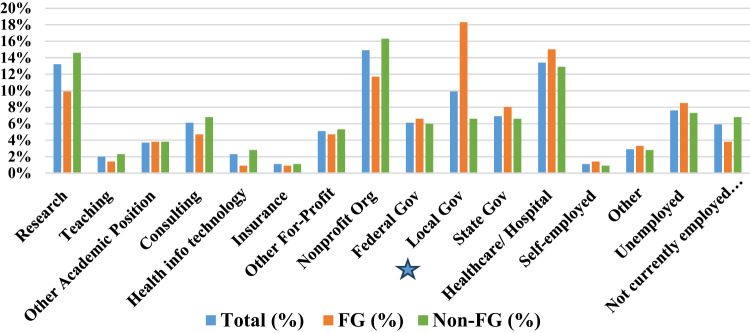
Job Sector of Employment by First Generation Status Star represents significant relationship from bivariate analysis

Figure 1 and Supplemental Table 1 provides a summary of the current job sectors of employment reported by participants stratified by education generation. The top three most common job sectors across the sample included non-profit organizations (14.9%), healthcare organization/hospital (13.4%), and research (13.2%). Among participants identifying as FG, the top three most common job sectors were local government (18.3%), healthcare organizations/hospitals (15.0%) and non-profit organizations (11.7%). Among non-FG participants, the three most common job sectors were non-profit organizations (16.3%), research (14.6%), and healthcare organization/hospital (12.9%). Significant differences in job sector of employment between FG and non-FG participants was observed for local government jobs (*p* < 0.001), with FG individuals having higher than expected representation in the sector.

**Table 2:** Job sector of employment by race/ethnic background (unweighted)

	Asian (*n* = 113)		Black (*n* = 102)		Hispanic (*n* = 80)		Other (*n* = 61)		White (*n* = 380)		Chi Square Test	Total (*n* = 751)	
	*n*	%	*n*	%	*n*	%	*n*	%	*n*	%	*p*-value	*n*	%
Research	19	16.8%	10	9.8%	13	16.3%	9	14.8%	44	11.6%	0.37	95	12.6%
Teaching	1	0.9%	2	2.0%	0	0.0%	1	1.6%	9	2.4%	0.74*	13	1.7%
Other Academic Position	3	2.7%	7	6.9%	5	6.3%	0	0.0%	11	2.9%	0.08*	26	3.5%
Consulting	9	8.0%	6	5.9%	3	3.8%	4	6.6%	23	6.1%	0.82*	45	6.0%
Health info technology	5	4.4%	1	1.0%	2	2.5%	1	1.6%	9	2.4%	0.61*	18	2.4%
Insurance	1	0.9%	1	1.0%	1	1.3%	2	3.3%	3	0.8%	0.36*	8	1.1%
Other For-Profit	5	4.4%	1	1.0%	2	2.5%	4	6.6%	21	5.5%	0.25*	33	4.4%
Nonprofit Org	10	8.8%	14	13.7%	11	13.8%	5	8.2%	67	17.6%	0.12	107	14.2%
Federal Gov	7	6.2%	7	6.9%	2	2.5%	7	11.5%	21	5.5%	0.25*	44	5.9%
Local Gov	6	5.3%	10	9.8%	16	20.0%	5	8.2%	37	9.7%	**0.02**	74	9.9%
State Gov	8	7.1%	4	3.9%	5	6.3%	3	4.9%	32	8.4%	0.65*	52	6.9%
Healthcare organization/Hospital	16	14.2%	18	17.6%	14	17.5%	7	11.5%	45	11.8%	0.39	100	13.3%
Self-employed	2	1.8%	1	1.0%	1	1.3%	1	1.6%	2	0.5%	0.37*	7	0.9%
Other	3	2.7%	0	0.0%	1	1.3%	2	3.3%	16	4.2%	0.19*	22	2.9%
Unemployed	11	9.7%	10	9.8%	1	1.3%	5	8.2%	30	7.9%	0.12*	57	7.6%
Not currently employed in PH	7	6.2%	10	9.8%	3	3.8%	5	8.2%	19	5.0%	0.30*	44	5.9%

*Computed with Fisher’s exact Chi-Square test

Table 2 provides an overview of job sector of employment among participants distributed across racial/ethnic backgrounds. Among Asian participants, the most common job sectors included research (16.8%) and healthcare organization/hospital (14.2%). For Black participants, working in healthcare organization/hospital (17.6%) and non-profit organizations (13.7%) were the most common job sectors of employment). Working in local government (20.0%) and healthcare organization/hospital (17.5%) were most common among Hispanic participants, while working in research (14.8%), the federal government (11.5%), and healthcare organization/hospital (11.5%) were the most common job sectors among Other participants. Lastly, among White participants, the most common job sectors of employment were non-profit organizations (17.65) and healthcare organization/hospital (11.8%). Significant differences in job sector of employment across racial and ethnic backgrounds was observed for local government (*p* ≤ 0.05) positions.

Lastly, Supplemental Table 2 provides an overview of job sector of employment among participants by gender identity. Among male participants, the most common job sectors of employment were in research (19.7%) and healthcare organization/hospital (15.4%) positions, while among female participants the most common job sectors of employment were with non-profit organizations (13.9%) and healthcare organization/hospital (12.9%). Among non-binary participants, the most common job sector of employment was in non-profit organizations (17.6%). Significant differences in job sector of employment by gender was observed for research jobs (*p* ≤ 0.05).

Further stratification of current job sectors by education generation status and gender revealed additional differences. For instance, FG male participants reported healthcare/hospital positions (21.3%) as the most common job sector of employment, whereas working in research was most common for non-FG males (22.9%). Similarly, FG female participants reported local government positions (19.8%) as the most common job sector of employment, while non-FG females reported working in non-profit organizations (15.4%) as the most common job sector of employment (Supplemental Table 3).

Similarly, when stratified by education generation status and race/ethnicity, other trends were observed. Employment in a healthcare organizations or hospitals was highest among Asian FG (18.2%), Black FG (15.8%), Black non-FG (18.8%), and Hispanic FG (16.3%) participants. Working in research positions was reported to be highest for Asian non-FG (17.5%), Hispanic non-FG (21.6%), and Other non-FG (18.6%) participants. White non-FG participants were more likely to work in nonprofit organizations (17.8%), while White FG participants were equally represented in nonprofit organizations and local government (16.3% each). Among Other FG participants, there was an equal percentage of individuals reported working in local government and healthcare/hospital settings (22.2% each) (Supplemental Table 4).

**Fig. 2:**
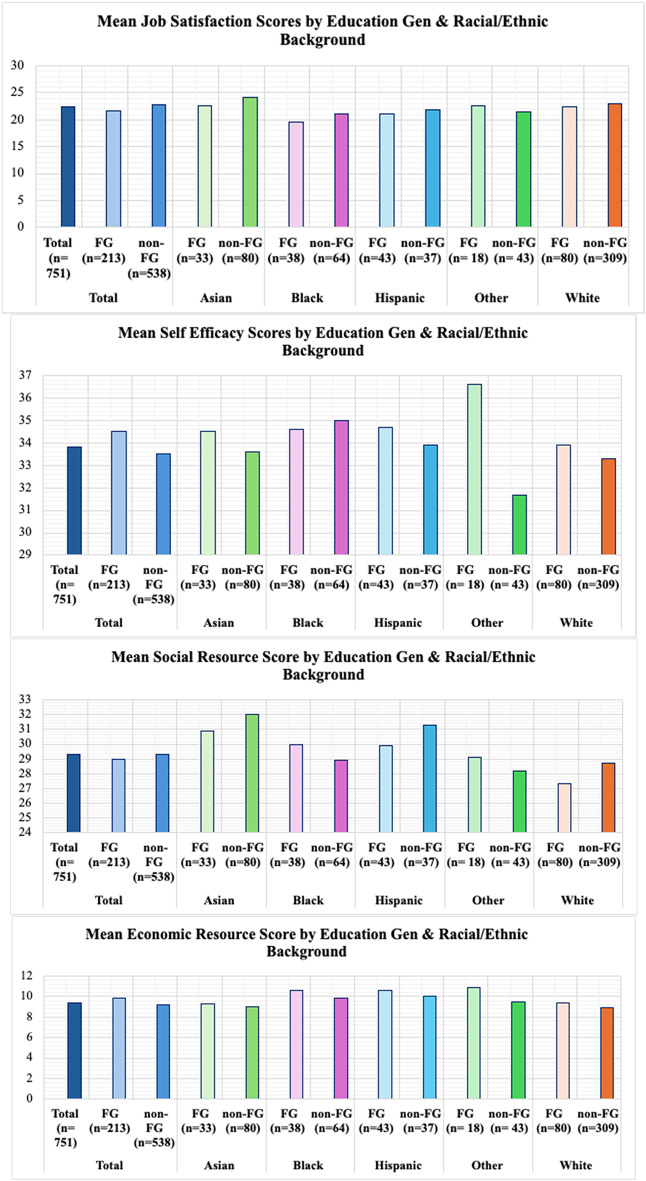
Distribution of Mean Job Satisfaction & Resource Scores by Race/Ethnicity and Education Generation Status

Figure 2 depicts average scores for job satisfaction and key resource domains across the full sample, with comparisons by race/ethnicity and education generation. On average, participants reported a job satisfaction score of 22.5 (range: 6–30), with FG participants scoring significantly lower (21.7) than their non-FG peers (22.8) (*p* < 0.05). The mean self-efficacy score was 33.8 (range: 15–40), with non-FG participants reporting significantly higher levels (34.5 vs. 33.5, *p* < 0.001) participants. For social resource scores, the average was 29.3 (range 13–55), with FG participants scoring similarly to their non-FG peers (29.0 vs. 29.3). Lastly, economic resource scores (mean: 9.4, range: 3–15) were also significantly higher among FG participants (9.9 vs. 9.2, *p* < 0.05). See Supplemental Table 5 for full tables and results.

When stratified by racial/ethnic background and education generation, significant differences were observed when comparing racial/ethnic groups to White participants (Supplemental Table 6). For instance, among FG individuals, there was a significant difference between average job satisfaction scores between Black FG and White FG individuals (19.6 vs. 22.4, *p* < 0.05). When examining mean self-efficacy scores, a significant difference was observed between Other FG and White FG individuals (36.6 vs. 33.9, *p* < 0.05). For social resource scores, significant differences were observed for Asian FG (30.9, *p* < 0.05), Black FG (30, *p* < 0.05), and Hispanic FG (29.9, *p* < 0.10) individuals when compared to mean scores for White FG participants (27.3). Lastly, significant differences in average economic resource scores were found for Black FG (10.6, *p* < 0.10), Hispanic FG (10.6, *p* < 0.05), and Other FG (10.9, *p* < 0.10) when compared to mean scores for White FG participants (9.35). The full table including differences between average scores between non-FG groups of color and White non-FG individuals can be found in Supplemental Materials (Supplemental Table 7).

### Associations between Resources and Job Satisfaction

Table 3 highlights the adjusted associations between resource domains on job satisfaction stratified separately by education generation status and race/ethnicity. Most resource domains were significant and positively associated with job satisfaction among both FG and non-FG participants, except for economic resources among non-FG individuals. The associations between self-efficacy and job satisfaction were stronger among non-FG as compared to their FG peers (β = 0.33, *p* < 0.001 vs. β = 0.23, *p* < 0.050). In contrast, stronger associations between social resources and job satisfaction were observed for FG participants (β = 0.35, *p* < 0.001) as compared to non-FG individuals (β = 0.22, *p* < 0.001). The association between economic resources and job satisfaction was only significant among the FG subgroup (β = 0.47, *p* < 0.05) and not among the non-FG subgroup.

Across racial/ethnic subgroups (Table 3), self-efficacy was significant and positively associated with job satisfaction among the Other (β = 0.42, *p* < 0.001), Asian (β = 0.38, *p* < 0.01), and White (β = 0.25, *p* < 0.01) subgroups. Social resources also demonstrated significant positive associations with job satisfaction across all racial/ethnic subgroups, with the strongest effect among those identifying as Black (β = 0.47, *p* < 0.001). Economic resources were only significantly associated with job satisfaction among those identifying as White (β = 0.31, *p* < 0.05) and Asian (β = 0.33, *p* < 0.05).

**Table 3:** Relationship of resources on job satisfaction (weighted, adjusted models)

	First-Generation (*n* = 213)		Non-First Generation (*n* = 538)		Asian (*n* = 113)		Black (*n* = 102)			Hispanic (*n* = 80)		Other (*n* = 43)		White (*n* = 389)	
Resource Type	β	95% CI	β	95% CI	β	95% CI		β	95% CI	β	95% CI	β	95% CI	β	95% CI
**Personal**															
*Self-efficacy*	0.23 *	0.01, 0.46	0.33 ***	0.14, 0.52	0.38 **	0.16, 0.60	0.16		−0.16, 0.47	0.28	−0.03, 0.60	0.42 **	0.15, 0.68	0.25 **	0.06, 0.43
**Social**															
*Social resource score*	0.35 ***	0.24, 0.45	0.22 ***	0.13, 0.32	0.20 ***	0.11, 0.30	0.47 ***		0.30, 0.64	0.32 ***	0.20, 0.43	0.18 *	0.01, 0.34	0.19 ***	0.09, 0.29
**Economic**															
*Econ resource score*	0.47 **	0.13, 0.80	−0.11	−0.37, 0.14	0.33 *	0.05, 0.60	0.17		−0.31, 0.65	0.20	−0.34, 0.74	−0.23	−0.60, 0.14	0.31 *	0.06, 0.56

Covariates for adjusted model include age, gender, marital status, age, degree type, financial, aid, first-generation status CI, confidence interval ***p < 0.001**p < 0.01*p < 0.05

**Fig. 3:**
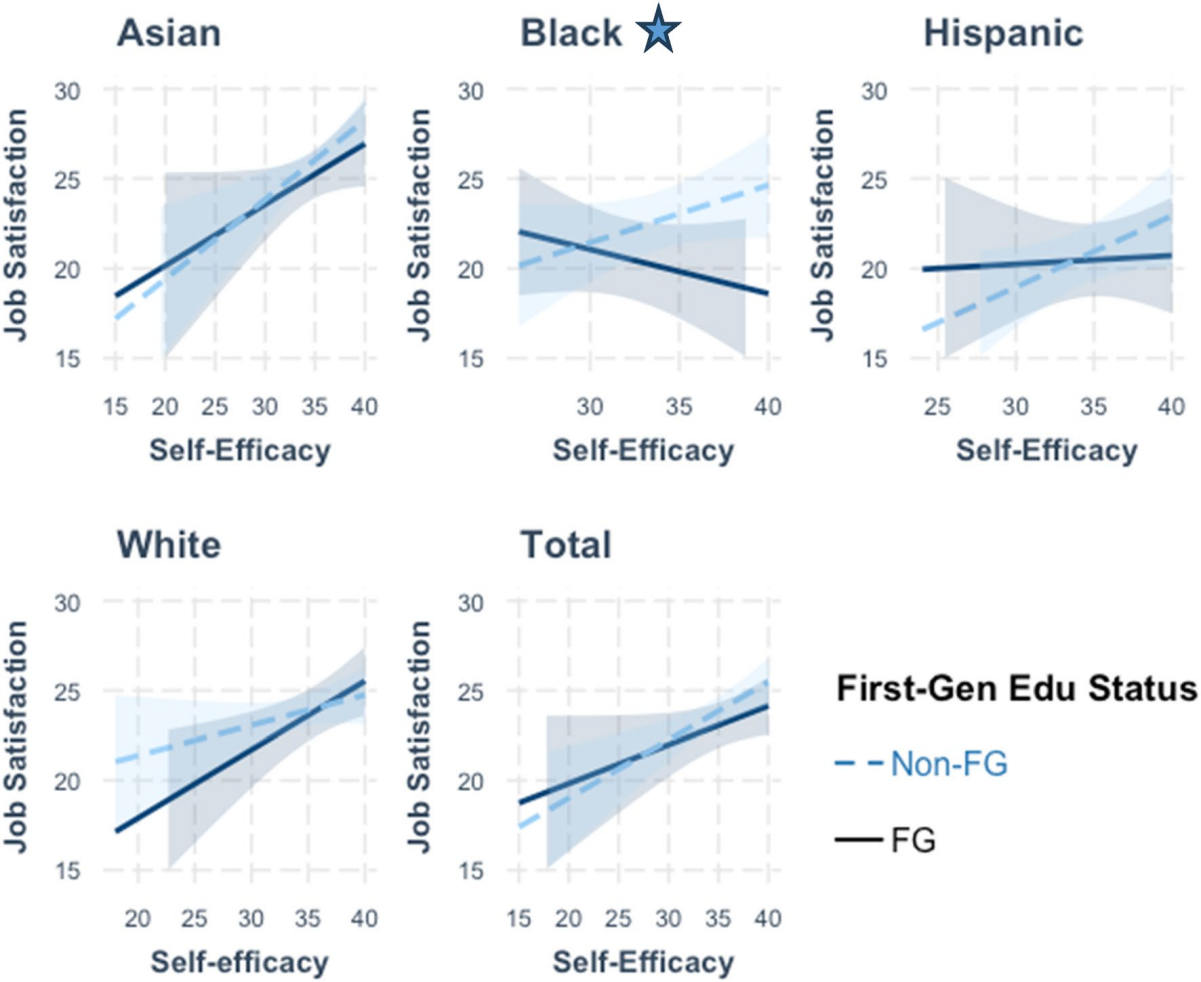
Interaction Effects of Self-Efficacy on Job Satisfaction by Racial/Ethnic Groups *Star represents significant interaction effect

**Fig. 4:**
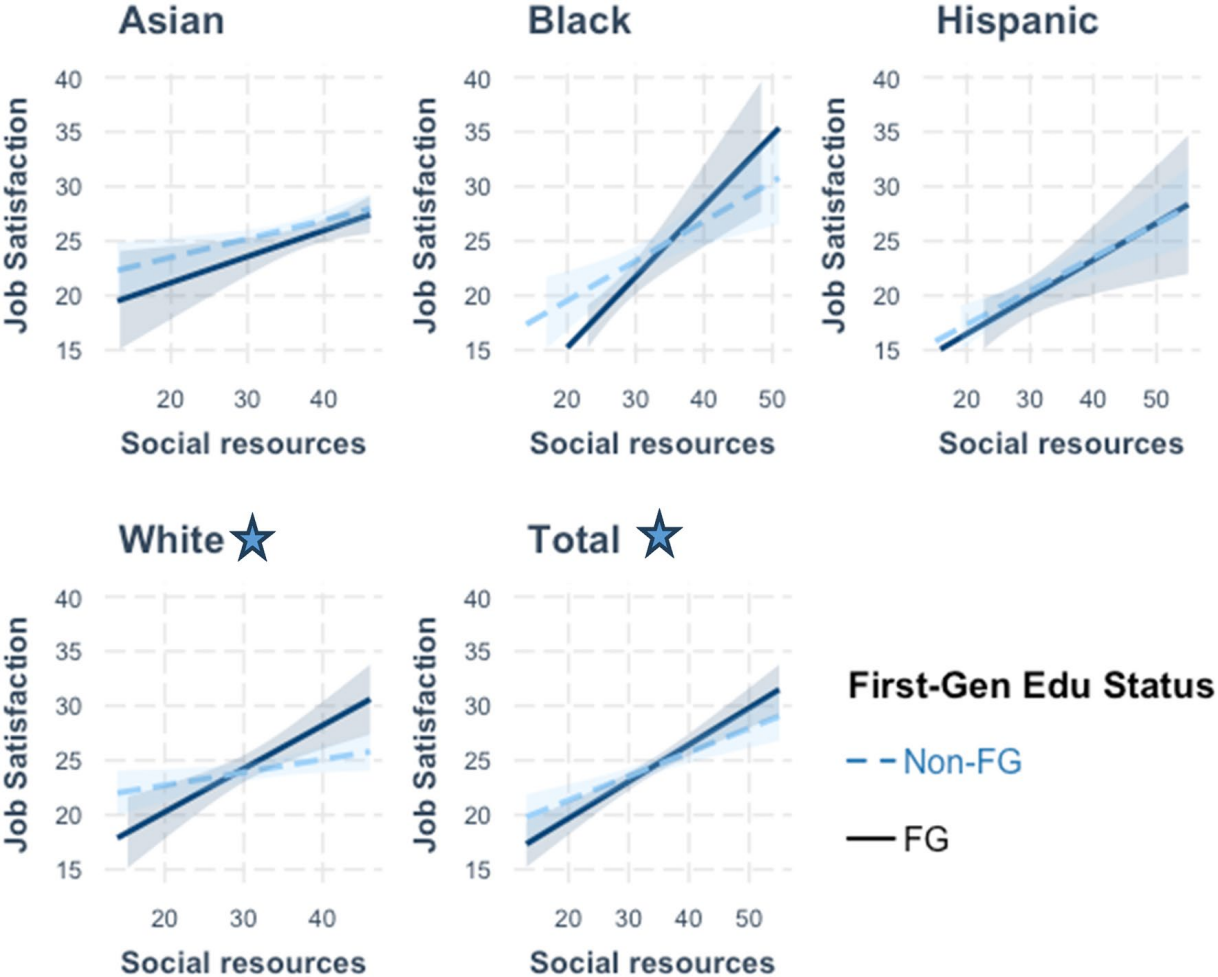
Interaction Effects of Social Resources on Job Satisfaction by Racial/Ethnic Groups *Star represents significant interaction effect

**Fig. 5:**
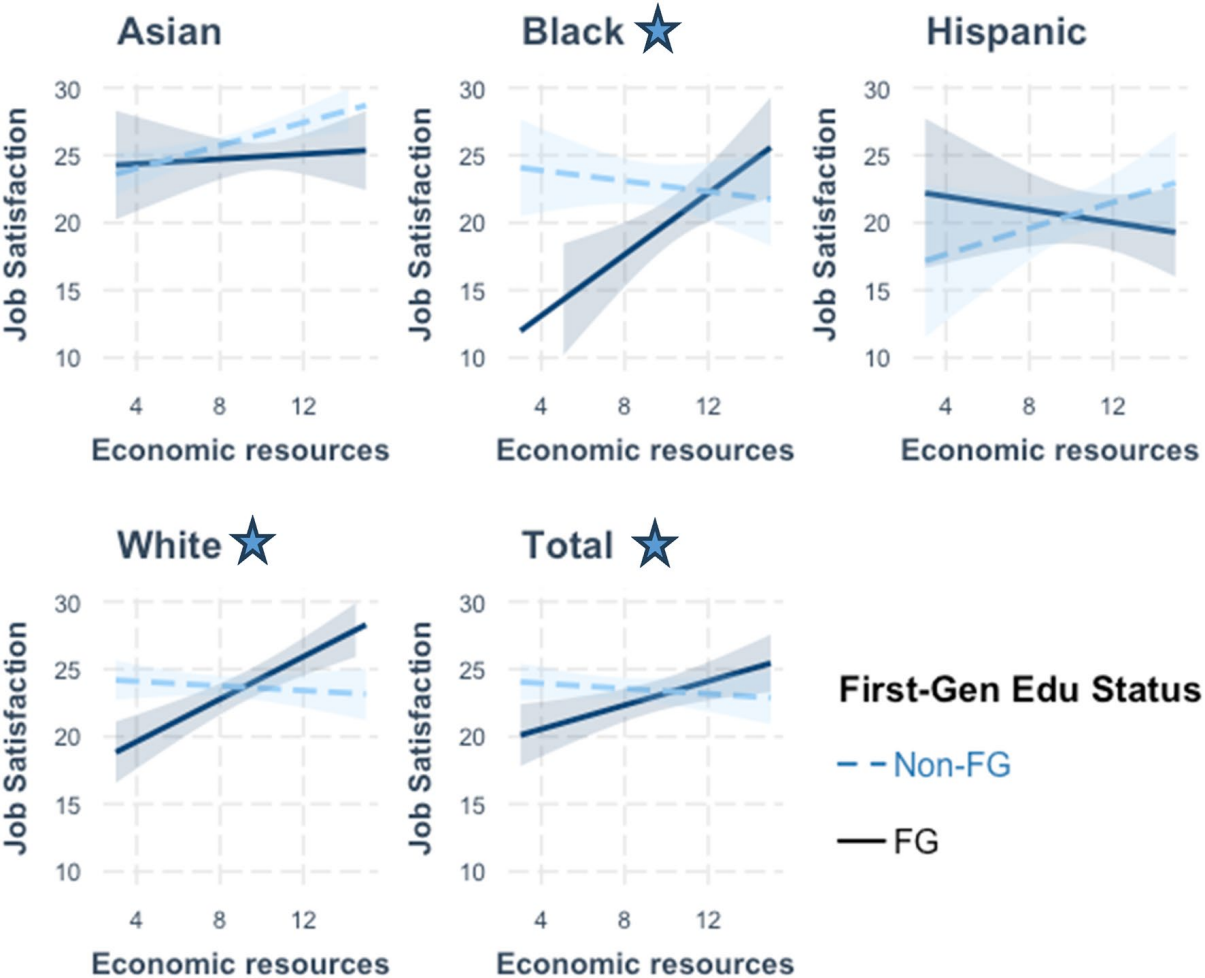
Interaction Effects of Economic Resources on Job Satisfaction by Racial/Ethnic Groups *Star represents significant interaction effect

Figures 3, 4 and 5 along with Supplemental Table 8 depict the results from examining more nuanced relationships by including the interaction effects of education generation status on the measures of personal, social, and economic resources when modelled for each racial/ethnic grouping. For personal resources, significant and positive associations were observed between self-efficacy scores and job satisfaction across the total study sample (β = 0.32, *p* < 0.01) and among the Asian (β = 0.44, *p* < 0.01) and Hispanic (β = 0.40 *p* < 0.05) subgroups for non-FG individuals. With measures of social resources, significant associations were observed with job satisfaction scores across the total sample (β = 0.22, *p* < 0.001) and among the Asian, Black, Hispanic, and White non-FG subgroups. Lastly, for economic resources, a significantly positive association with job satisfaction was observed for the Asian non-FG subgroup (β = 0.42, *p* < 0.01).

Further examination of the relationship between FG status on job satisfaction revealed notable subgroup differences. Among participants identifying as Black, FG status was marginally associated with higher job satisfaction in self-efficacy models (β = 16.59, *p* < 0.10), but was negatively associated with job satisfaction for both social (β= −10.02, *p* < 0.10) and economic (β= −16.07, *p* < 0.01) resources. Among participants identifying as White, FG status was negatively associated with job satisfaction in models for social (β= −8.04, *p* < 0.01) and economic (β= −7.96, *p* < 0.001) resources. Across the full sample, FG status demonstrated a significant and negative association with job satisfaction in models for social (β= −4.00, *p* < 0.10) and economic (β = −5.56, *p* < 0.01) resources (ST 8, Models 2 & 3).

When delving into interaction effects, relationships between resources and job satisfaction were found to differ when moderated with FG status. Significant interaction effects were found for self-efficacy scores among those identifying as Black (β =−0.57, *p* < 0.10) (ST 8, Model 1; Fig. 3). Among Black respondents, self-efficacy showed a positive association with job satisfaction (β = 0.32) for non-FG individuals, whereas for FG individuals, the slope was negative (β =−0.25). This suggests that increases to self-efficacy may not lead to higher job satisfaction for Black FG students.

From Fig. 4, the interaction effects between FG status and social resources were statistically significant among participants in the White (β = 0.28, *p* < 0.01) subgroup and across the entire study sample (β = 0.12, *p* < 0.10). In the full sample, social resources were positively associated with job satisfaction for both non-FG (β = 0.22, *p* < 0.001) and FG individuals, with stronger effects among FG individuals (β = 0.34), particularly among White respondents, where the association was higher for FG respondents (β = 0.40). As shown in Fig. 5, the interaction effects between FG status and economic resources on job satisfaction were statistically significant among participants who identified as Black (β = 1.33, *p* < 0.01), White (β = 0.87, *p* < 0.001), and across the total sample (β = 0.54, *p* < 0.01), suggesting that seeking jobs based on financial requirements may help to offset some of the negative effects of FG status on job satisfaction. For the full sample, economic resources were not related to job satisfaction for non-FG individuals, whereas a positive moderating effect of increased economic resources on job satisfaction was found for FG individuals (β = 0.44).

Table 4 presents final models after removing non-significant interaction terms. In these models, self-efficacy was significantly and positively associated with job satisfaction across all groups (Table 4, Model 1). In Model 2, social resources were significant and positively associated with job satisfaction among the Asian (β = 0.20, *p* < 0.001), Hispanic (β = 0.32, *p* < 0.001), and Black (β = 0.47, *p* < 0.001) subgroups. Furthermore, first-generation status was significant and negatively associated for the Asian subgroup (β= −1.31, *p* < 0.05). Similarly, among the Asian subgroup, a significantly positive association between economic resource and job satisfaction was observed (β = 0.33, *p* < 0.05).

**Table 4:** Final models removing Non-Significant interaction effects by racial/ethnic groups (weighted, adjusted models)

	Model 1: Personal Resources									
	Asian (*n* = 113)		Black (*n* = 102)		Hispanic (*n* = 80)		White (*n* = 389)		Total (*n* = 751)	
	**β**	95% CI	**β**	95% CI	**β**	95% CI	**β**	95% CI	**β**	95% CI
Self-efficacy	0.38**	0.16, 0.60			0.28.	−0.03, 0.60	0.25**	0.06, 0.43	0.28***	0.14, 0.43
First-Generation Status	−0.74	−2.27, 0.80			−0.34	−2.89, 2.21	−0.59	−2.56, 1.38	−0.69	−2.30, 0.92
	**Model 2: Social Resources**									
	Asian		Black		Hispanic		White		Total	
	**β**	95% CI	**β**	95% CI	**β**	95% CI	**β**	95% CI	**β**	95% CI
Social Resources	0.20***	0.11, 0.30	0.47***	0.30, 0.64	0.32***	0.20, 0.43				
First-Generation Status	−1.31*	−2.58, −0.04	−1.71	−3.92, 0.50	−0.5	−2.72, 1.72				
	**Model 3: Economic Resources**									
	Asian		Black		Hispanic		White		Total	
	**β**	95% CI	**β**	95% CI	**β**	95% CI	**β**	95% CI	**β**	95% CI
Economic Resources	0.33*	0.05, 0.60			0.20	−0.34, 0.74				
First-Generation Status	−1.34	−2.69, 0.01			−0.31	−2.96, 2.34				

***p < 0.001**p < 0.01*p < 0.05Covariates for adjusted model include age, gender, marital status, age, degree type, financial, aid, first-generation statusCI, confidence interval

Table 5 depicts models with significant interaction effects were further stratified by race/ethnic group and education generation status. Since no significant interaction effects were observed across the three resource domains for the Asian and Hispanic subgroups, they were omitted from the table to enhance clarity in data presentation. From Model 1, a no significant association was observed among the Black FG and Black non-FG subgroups. In Model 2, social resources were significant and positively associated with job satisfaction across White FG (β = 0.36, *p* < 0.001) and across the total FG (β = 0.35, *p* < 0.001) and total non-FG (β = 0.22, *p* < 0.001) groups. Notably, the strengths of these associations were slightly greater among the FG subgroups as compared to the non-FG subgroups (Model 2). Lastly, in Model 3, economic resources were found to be significant and positively associated with job satisfaction among Asian non-FG (β = 0.33, *p* < 0.05), Black FG (β = 0.80, *p* < 0.05), White FG (β = 0.62, *p* < 0.001) and across all FG (β = 0.47, *p* < 0.01) individuals.

**Table 5:** Final models to parse significant interaction effects by race/ethnicity & education generation (weighted, adjusted models)

	Model 1: Personal Resources																								
		Black FG		Black NFG			White FG			White NFG					Total FG				Total non-FG						
		**β** 95% CI		**β** 95% CI			**β** 95% CI					**β** 95% CI				**β** 95% CI						**β** 95% CI			
Self-efficacy		−0.45 (−0.9, 0.02)		0.21 (−0.1, 0.5)																					
	**Model 2: Social Resources**																								
	Black FG		Black NFG			White FG			White NFG					Total FG						Total non-FG					
	**β** 95% CI			**β** 95% CI		**β** 95% CI						**β** 95% CI					**β** 95% CI							**β** 95% CI	
Soc Res					0.36*** (0.2, 0.5)						0.1 (−0.001, 0.2)			0.35*** (0.2, 0.5)										0.22*** (0.1, 0.3)	
	**Model 3: Economic Resources**																								
	Black FG		Black NFG			White FG			White NFG					Total FG						Total non-FG					
	**β** 95% CI			**β** 95% CI		**β** 95% CI						**β** 95% CI				**β** 95% CI					**β** 95% CI				
Econ Res	0.8* (0.1, 1.5)		−0.08 (−0.6, 0.4)		0.62*** (0.3, 0.9)						−0.05 (−0.3, 0.2)			0.47** (0.1, 0.8)									−0.11 (−0.4, 0.10)		

**p < 0.01*p < 0.05Covariates for adjusted model include age, gender, marital status, age, degree type, financial, aid, first-generation statusCI, confidence interval

## Discussion

In this study, we analyzed survey data from master’s and doctoral-level public health graduates to examine how personal, social, and economic resources influence job satisfaction across FG and non-FG graduates and by race/ethnicity. Our findings suggest that all three resource domains may be important for first-generation graduates- namely FG graduates of color- and strengthening these domains may improve job satisfaction outcomes for this population. Our findings have important implications for both public health academic institutions and employers seeking to engage meaningfully with diverse graduates entering the workforce.

We found minimal differences in job sector selection by FG status. However, FG graduates were significantly more likely to work in local government positions compared to their non-FG peers. Although research on job sector trends by education generation status is limited, local government roles may offer financial stability through benefits such as retirement plans, and rising salaries created by an aging and retiring workforce [44, 45]. While limited data exists on the financial circumstances of FG graduate students, evidence shows that FG undergraduate students are more likely to have student loan debt compared to their non-FG peers [46]. Data from the National Center for Education Statistics (2018–2019) estimated the median debt for completing a master’s level public health degree was $44,479, and $74, 837 for completing a doctorate level degree [47]. It is likely that FG graduate students will continue to rely on student loans to fund their education and may select careers that enable them to repay these debts [48].

When examining trends by intersecting education generation and racial/ethnic identities, non-White FG subgroups reported working in healthcare or hospital settings (Asian FG, Black FG, Hispanic FG, Other FG) more than White FG and White non-FG subgroups. This suggests that job sector selection for public health graduates of master’s and doctoral level degrees may be influenced by education generation status and racial/ethnic identities. For FG individuals from racial and ethnically minoritized backgrounds, jobs in healthcare or hospital settings may offer advantages and appeal to both serve the community’s needs with professional stability. In contrast, non-FG individuals from racial and ethnically minoritized backgrounds may pursue research based on their networks and professional mentorship, whether developed through their own personal ties or familial connections [49].

It is interesting to note that White FG and non-FG participants were more likely to work in public health related non-profit settings, which may be attributed to structural factors such as advantages White workers have compared to non-White workers in accessing career advancement in non-profit positions through resources such as mentorship, professional development, and executive coaching [50]. In their report based on a survey of more than 5,000 non-profit staff, respondents of color identified several systemic barriers as obstacles to working in the non-profit sector generally, including inadequate and inequitable salaries, few opportunities for advancement, lack of role models, lack of relationships with funders, lack of social capital/networks, and the burdens of being called to represent a community and to “push DEI efforts” [50]. Therefore, working in the non-profit sector for public health may be perceived differently across populations and may also integrate racial and ethnic groups inequitably into organizational roles and opportunities.

Our results illustrate how each resource domain (personal, social, and economic) influences job satisfaction among public health master’s and doctoral level graduates, and how results differed across race/ethnicity and FG status. Personal resources, measured by self-efficacy, were positively associated with job satisfaction overall, with stronger effects among non-FG as compared to FG participants. Overall, our findings suggest that supporting self-efficacy among all graduate students may be important in their transition into the workforce. Furthermore, FG status was observed to negatively moderate the relationship between self-efficacy and job satisfaction among Black identifying individuals, suggesting that increased self-efficacy may not necessarily translate to higher job satisfaction for this group. This relationship may reflect the systemic barriers that Black FG individuals encounter in when navigating layers of systemic barriers, both for their education and transition into the public health workforce [51].

Strategies to promote self-efficacy among graduate students have included recommendations for academic programs to provide emotional and academic support, both with academic supervisors as well as through cohort based approaches to address barriers and support students in successfully completing their degrees [52]. Future research should assess additional measures of self-efficacy (i.e.: academic self-efficacy, career self-efficacy, financial self-efficacy) among FG public health graduate students of color and explore how an emphasis on individual agency realistically interacts with the challenges of navigating structural barriers [51, 53]. These findings could inform the development and evaluation of pathway programs tailored to support first-generation public health graduate students of color in successfully matriculating through and completing master’s and doctoral programs.

Measures of social resources were significant and positively associated with levels of job satisfaction after controlling for covariates across the entire sample. This is consistent with previous research into the experience of FG undergraduate students, where social resources in the form of support from family, friends, and significant others have been shown to lead to a greater likelihood of positive career outcome expectations [54]. Non-FG students would have access to social resources through intergenerational knowledge, providing information on navigating the costs and benefits of investing in higher education and career decisions through family members’ experiences [55].

Additionally, social resources were found to be significant and positively associated with job satisfaction among all racial/ethnic subgroups from our study results. Furthermore, FG status also significantly moderated the relationship of social resources on job satisfaction in the total sample and among the White subgroup in our study. While positive associations between social resources on job satisfaction were observed for both FG and non-FG White and total subgroups, the effects were stronger among the FG groupings. This suggests that while social resources are important for both FG and non-FG individuals during their job searches, there may be a more pronounced impact on job satisfaction for FG individuals. These findings reflect the role social resources (e.g., social networks) may play in mentorship and career advancement, with more attention needed to explore nuances of these dynamics among minoritized populations where associations with job satisfaction were stronger [56].

Lastly, significant associations of economic resources on job satisfaction were observed among Black FG, White FG, and the total FG groupings. This finding aligns with prior research on FG college graduates transitioning to the workplace, which found that financial factors, such as student loan obligations and a desire for greater financial stability, played a critical role in shaping their job choices [48]. FG status also significantly moderated the relationship between economic resources and job satisfaction among the full sample, as well as for individuals identifying as Black and White, with stronger effects for individuals identifying as Black. These associations were more also pronounced among Black FG, White FG, and across the total FG subgroups (with the highest effect observed for Black FG individuals), suggesting that economic resources, and the importance of meeting financial needs, may play a more influential role in shaping job satisfaction for FG graduates and FG graduates of color as compared to non-FG graduates.

There are several limitations to note for this study. First, because the survey was anonymous and distributed through academic and professional networks, it was not possible to calculate an accurate response rate. As a result, there is potential for nonresponse and self-selection bias, as the findings reflect only those who chose to participate. The survey link was shared via LinkedIn and Twitter, and given the need to protect anonymity, the research team was unable to track all individuals who clicked on the link and whether they completed the survey or not to obtain a response rate. Therefore, sampling bias is a concern due to the use of convenience sampling. Although the survey period was extended to support broader recruitment, achieving a representative sample proved difficult. To address sampling bias, a post-survey weight was created using data from the 2021 National Science Foundation’s National Survey of College Graduates.

Although efforts were made to oversample underrepresented populations, aggregation was still necessary for certain ethnic/racial identities due to limited sample sizes. Future research should prioritize oversampling from Native American/American Indian, Pacific Islander/Native Hawaiian, Middle Eastern & North African, and multiracial identities to more accurately capture and highlight the experiences of these populations. Disaggregating the “Other” category and collecting data by ethnicity will be important to uncover additional relationships. Similarly, given the low representation of non-binary individuals, gender was measured as a binary variable for model development. Future studies should consider oversampling underrepresented gender identity intersections to better capture the experiences of marginalized groups, while also incorporating qualitative methods to deepen understanding of the nuanced experiences of underrepresented public health graduate student populations.

Furthermore, the use of self-reported survey data introduces bias, including recall and social desirability bias. To improve reliability and comparability in future research, incorporating validated measures of job satisfaction is recommended. While this study did not directly examine broader structural influences, such as systemic discrimination in hiring, recruitment, or job performance, these factors likely shape the workplace experiences of minoritized groups in public health. Further research is needed to investigate how both individual-level experiences and structural inequities affect career trajectories and job satisfaction among diverse public health professionals.

Lastly, this study was not able to examine other key factors such as mental and physical health, stress, perceived career growth and opportunities, work relationships, coping mechanisms, geographic location, academic performance, student debt load, and other socioeconomic data. There are limitations to the generalizability of our findings which should be interpreted with caution, particularly when considering their applicability to populations beyond the study sample. To address the limitations inherent in the cross-sectional design of this study, future research should consider alternative designs, such as a cohort study following public health students in the final year of their graduate-level training and subsequent transition into the workforce. Despite these limitations, the study offers useful insights that can inform and guide future national research efforts focusing on public health master’s and doctoral level graduates.

**Fig. 6:**
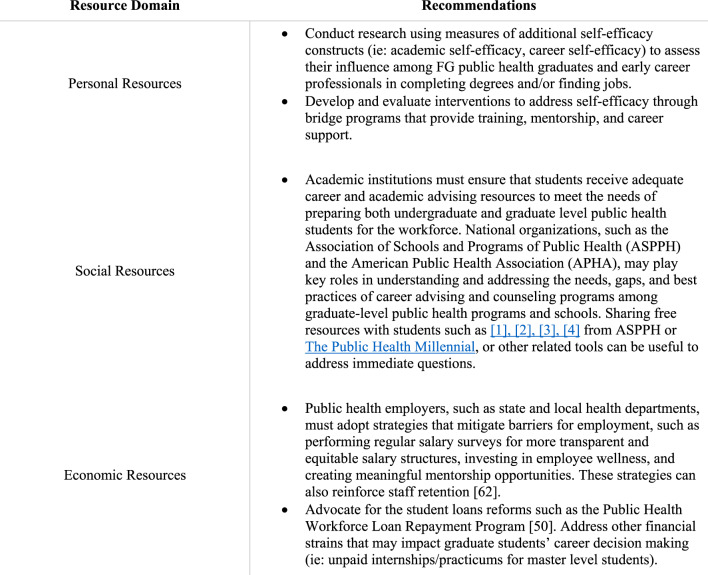
Resource related recommendations for public health institutions

Our findings highlight the need for stronger collaboration between public health employers and academic institutions to address the barriers, priorities, and aspirations of public health graduate students, particularly FG graduate students of color. Structural changes are required of all institutions to establish supportive pathways for students’ successful transitions into the public health workforce. Below in Fig. 6, we highlight opportunities for enhancing resource domains. Based on our findings, identifying strategies that strengthen personal, social, and economic resources for public health FG graduate students has the potential to promote success for all students to contribute to public health infrastructure and systems.

## Conclusion

This study is among the first to examine how levels of personal, social, and economic resources influence job satisfaction, and how these experiences differ across intersecting identities of race/ethnicity and education generation status. Our findings suggest that these resources may play a role in mitigating lower levels of job satisfaction among FG public health professionals in their transition into the workforce. Given the associations observed between resource measure scores and job satisfaction among FG and racially/ethnically diverse subgroups, future efforts to support graduate-level students’ transition into the public health workforce should more intentionally address how these resources influence career decision making and job satisfaction. Multi-pronged strategies that cultivate graduate students’ self-efficacy, strengthen access to career advising and influential social networks, and reduce economic barriers are critical for improving workforce preparation and stability. By integrating these practices, public health institutions can contribute towards cultivating a more diverse, resilient, and culturally responsive workforce.

## Supplementary Information

Below is the link to the electronic supplementary material.

Supplementary Material 1

## Data Availability

The dataset created and/or analyzed during the current study is available from the corresponding author upon reasonable request.
