# Supplementary material for: The Role of Resources on Job Satisfaction among US Public Health Master’s and Doctoral Program Graduates at the Intersection of Race, Ethnicity, and First-Generation Status
Source: J Community Health. 2025 Sep 11;51(1):69–86. doi: 10.1007/s10900-025-01513-2 (PMC12950058; doi:10.1007/s10900-025-01513-2)
Supplement: Supplementary file 1 — Supplementary Material 1 [file 10900_2025_1513_MOESM1_ESM.docx]

**Supplemental Materials**

**The Role of Resources on Job Satisfaction among US Public Health Master’s and Doctoral Program Graduates at the Intersection of Race, Ethnicity, and First-Generation Status**

Kimberly Wu, MPH^1*^; Felicia Setiono, PhD^2^; W. Marcus Lambert, PhD^2^; Shokufeh Ramirez, PhD, MPH^1^; Christine M. Arcari, PhD, MPH^3^; Katherine P. Theall, PhD, MPH^1^; Dovile Vilda, PhD, MSc^1^

^1^ Department of Social, Behavioral, and Population Sciences, Celia Scott Weatherhead School of Public Health and Tropical Medicine, New Orleans, LA 70112, USA

^2^ SUNY Downstate Health Sciences University, Brooklyn, NY 11203, USA

^3^ Department of Epidemiology, Celia Scott Weatherhead School of Public Health and Tropical Medicine, New Orleans, LA 70112, USA

**Corresponding Author:**

**Kimberly Wu, MPH**

Doctoral Candidate

Department of Social, Behavioral, and Population Sciences

Tulane University School of Public Health and Tropical Medicine

New Orleans, LA 70112, USA

Email: [Kwu6@tulane.edu](mailto:Kwu6@tulane.edu)

ORCID: <https://orcid.org/0000-0003-0638-3199>

**Journal**: Research in Higher Education

**Supplemental Figure 1. DAG**


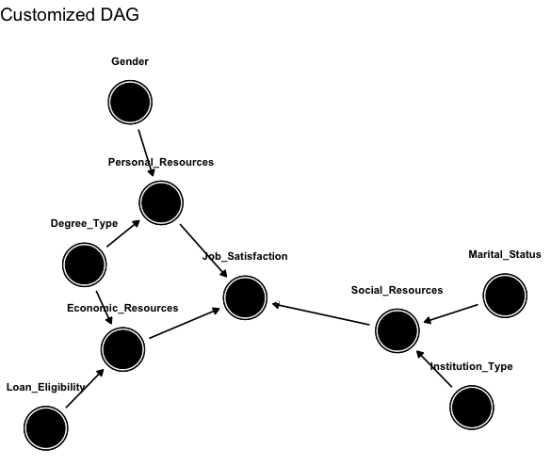


**Supplemental Table 1. Job Sector of Employment of Study Participants by Education Generation Status (unweighted)**

|  | **Total (n=751)** | | **First-Generation (n=213)** | | **Non-First Generation (n=538)** | | **Chi-Square Test** |
| --- | --- | --- | --- | --- | --- | --- | --- |
|  | **n** | **%** | **n** | **%** | **n** | **%** | **p-value** |
| Research | 99 | 13.2% | 21 | 9.9% | 78 | 14.6% | 0.18 |
| Teaching | 15 | 2.0% | 3 | 1.4% | 12 | 2.3% | 0.77* |
| Other Academic Position | 28 | 3.7% | 8 | 3.8% | 20 | 3.8% | 0.29* |
| Consulting | 46 | 6.1% | 10 | 4.7% | 36 | 6.8% | 0.40* |
| Health info technology | 17 | 2.3% | 2 | 0.9% | 15 | 2.8% | 0.12* |
| Insurance | 8 | 1.1% | 2 | 0.9% | 6 | 1.1% | 1.00 |
| Other For-Profit | 38 | 5.1% | 10 | 4.7% | 28 | 5.3% | 0.70* |
| Nonprofit Org | 112 | 14.9% | 25 | 11.7% | 87 | 16.3% | 0.26 |
| Federal Gov | 46 | 6.1% | 14 | 6.6% | 32 | 6.0% | 0.86 |
| Local Gov | 74 | 9.9% | 39 | 18.3% | 35 | 6.6% | **<0.001** |
| State Gov | 52 | 6.9% | 17 | 8.0% | 35 | 6.6% | 0.52* |
| Healthcare organization/ Hospital | 101 | 13.4% | 32 | 15.0% | 69 | 12.9% | 0.56 |
| Self-employed | 8 | 1.1% | 3 | 1.4% | 5 | 0.9% | 0.69* |
| Other | 22 | 2.9% | 7 | 3.3% | 15 | 2.8% | 0.81* |
| Unemployed | 57 | 7.6% | 18 | 8.5% | 39 | 7.3% | 0.68 |
| Not currently employed in PH | 44 | 5.9% | 8 | 3.8% | 36 | 6.8% | 0.12 |
| *Computed with Fisher's exact Chi-Square test | | | | | | | |

| \| **Supplemental Table 2. Job Sector of Employment of Study Participants by Gender (unweighted)** \| \| \| \| \| \| \| \| \| \| \| --- \| --- \| --- \| --- \| --- \| --- \| --- \| --- \| --- \| --- \| \|  \| **Male (n=117)** \| \| **Female**  **(n= 627)** \| \| **Non-binary (n=17)** \| \| **Chi Square Test** \| **Total (n=751)** \| \| \|  \| **n** \| **%** \| **n** \| **%** \| **n** \| **%** \| **p-value** \| **n** \| **%** \| \| Research \| 23 \| 19.7% \| 70 \| 11.2% \| 2 \| 11.8% \| **0.05*** \| 95 \| 12.6% \| \| Teaching \| 2 \| 1.7% \| 12 \| 1.9% \| 0 \| 0.0% \| 1* \| 14 \| 1.9% \| \| Other Academic Position \| 2 \| 1.7% \| 25 \| 4.0% \| 0 \| 0.0% \| 0.42* \| 27 \| 3.6% \| \| Consulting \| 4 \| 3.4% \| 40 \| 6.4% \| 2 \| 11.8% \| 0.20* \| 46 \| 6.1% \| \| Health info technology \| 4 \| 3.4% \| 14 \| 2.2% \| 0 \| 0.0% \| 0.68* \| 18 \| 2.4% \| \| Insurance \| 1 \| 0.9% \| 7 \| 1.1% \| 0 \| 0.0% \| 1* \| 8 \| 1.1% \| \| Other For-Profit \| 5 \| 4.3% \| 28 \| 4.5% \| 0 \| 0.0% \| 1* \| 33 \| 4.4% \| \| Nonprofit Org \| 17 \| 14.5% \| 87 \| 13.9% \| 3 \| 17.6% \| 0.85* \| 107 \| 14.2% \| \| Federal Gov \| 8 \| 6.8% \| 34 \| 5.4% \| 2 \| 11.8% \| 0.32* \| 44 \| 5.9% \| \| Local Gov \| 10 \| 8.5% \| 63 \| 10.0% \| 1 \| 5.9% \| 0.92* \| 74 \| 9.9% \| \| State Gov \| 1 \| 0.9% \| 45 \| 7.2% \| 1 \| 5.9% \| 0.79* \| 47 \| 6.3% \| \| Healthcare organization/ Hospital \| 18 \| 15.4% \| 81 \| 12.9% \| 1 \| 5.9% \| 0.62* \| 100 \| 13.3% \| \| Self-employed \| 0 \| 0.0% \| 8 \| 1.3% \| 0 \| 0.0% \| 0.47* \| 8 \| 1.1% \| \| Other \| 1 \| 0.9% \| 20 \| 3.2% \| 1 \| 5.9% \| 0.20* \| 22 \| 2.9% \| \| Unemployed \| 9 \| 7.7% \| 46 \| 7.3% \| 2 \| 11.8% \| 0.65* \| 57 \| 7.6% \| \| Not currently employed in PH \| 7 \| 6.0% \| 37 \| 5.9% \| 2 \| 11.8% \| 0.49* \| 46 \| 6.1% \| \| *Computed with Fisher's exact Chi-Square test \| \| \| \| \| \| \| \| \| \|   **Supplemental Table 3. Job Sector of Employment by Gender and Education Generation** | | | | | | | | | | |
| --- | --- | --- | --- | --- | --- | --- | --- | --- | --- | --- | --- | --- | --- | --- | --- | --- | --- | --- | --- | --- | --- | --- | --- | --- | --- | --- | --- | --- | --- | --- | --- | --- | --- | --- | --- | --- | --- | --- | --- | --- | --- | --- | --- | --- | --- | --- | --- | --- | --- | --- | --- | --- | --- | --- | --- | --- | --- | --- | --- | --- | --- | --- | --- | --- | --- | --- | --- | --- | --- | --- | --- | --- | --- | --- | --- | --- | --- | --- | --- | --- | --- | --- | --- | --- | --- | --- | --- | --- | --- | --- | --- | --- | --- | --- | --- | --- | --- | --- | --- | --- | --- | --- | --- | --- | --- | --- | --- | --- | --- | --- | --- | --- | --- | --- | --- | --- | --- | --- | --- | --- | --- | --- | --- | --- | --- | --- | --- | --- | --- | --- | --- | --- | --- | --- | --- | --- | --- | --- | --- | --- | --- | --- | --- | --- | --- | --- | --- | --- | --- | --- | --- | --- | --- | --- | --- | --- | --- | --- | --- | --- | --- | --- | --- | --- | --- | --- | --- | --- | --- | --- | --- | --- | --- | --- | --- | --- | --- | --- | --- | --- | --- | --- | --- | --- | --- | --- | --- | --- | --- | --- | --- | --- | --- | --- | --- | --- | --- | --- | --- | --- | --- | --- | --- | --- | --- | --- | --- | --- | --- | --- |
|  | **Male FG (n=47)** | | **Male non-FG (n=70)** | | **Female FG (n= 162)** | | **Female non-FG (n=455)** | | **Total (n=751)** | |
|  | **n** | **%** | **n** | **%** | **n** | **%** | **n** | **%** | **n** | **%** |
| Research | 7 | 14.9% | 16 | **22.9%** | 14 | 8.6% | 56 | 12.3% | 93 | 12.4% |
| Teaching | 0 | 0.0% | 2 | 2.9% | 3 | 1.9% | 9 | 2.0% | 14 | 1.9% |
| Other Academic Position | 1 | 2.1% | 1 | 1.4% | 4 | 2.5% | 21 | 4.6% | 27 | 3.6% |
| Consulting | 0 | 0.0% | 4 | 5.7% | 8 | 4.9% | 32 | 7.0% | 44 | 5.9% |
| Health info technology | 0 | 0.0% | 4 | 5.7% | 2 | 1.2% | 12 | 2.6% | 18 | 2.4% |
| Insurance | 0 | 0.0% | 1 | 1.4% | 2 | 1.2% | 5 | 1.1% | 8 | 1.1% |
| Other For-Profit | 1 | 2.1% | 4 | 5.7% | 7 | 4.3% | 21 | 4.6% | 33 | 4.4% |
| Nonprofit Org | 7 | 14.9% | 10 | 14.3% | 17 | 10.5% | 70 | **15.4%** | 104 | **13.8%** |
| Federal Gov | 5 | 10.6% | 3 | 4.3% | 7 | 4.3% | 27 | 5.9% | 42 | 5.6% |
| Local Gov | 7 | 14.9% | 3 | 4.3% | 32 | **19.8%** | 31 | 6.8% | 73 | 9.7% |
| State Gov | 4 | 8.5% | 2 | 2.9% | 13 | 8.0% | 32 | 7.0% | 51 | 6.8% |
| Healthcare organization/ Hospital | 10 | **21.3%** | 8 | 11.4% | 22 | 13.6% | 59 | 13.0% | 99 | 13.2% |
| Self-employed | 0 | 0.0% | 0 | 0.0% | 3 | 1.9% | 5 | 1.1% | 8 | 1.1% |
| Other | 1 | 2.1% | 0 | 0.0% | 6 | 3.7% | 14 | 3.1% | 21 | 2.8% |
| Unemployed | 4 | 8.5% | 5 | 7.1% | 14 | 8.6% | 32 | 7.0% | 55 | 7.3% |
| Not currently employed in PH | 0 | 0.0% | 7 | 10.0% | 8 | 4.9% | 29 | 6.4% | 44 | 5.9% |
| *Computed with Fisher's exact Chi-Square test | | | | | | |  |  |  |  |

**Supplemental Table 4. Job Sector of Employment by Race/Ethnicity and Education Generation**


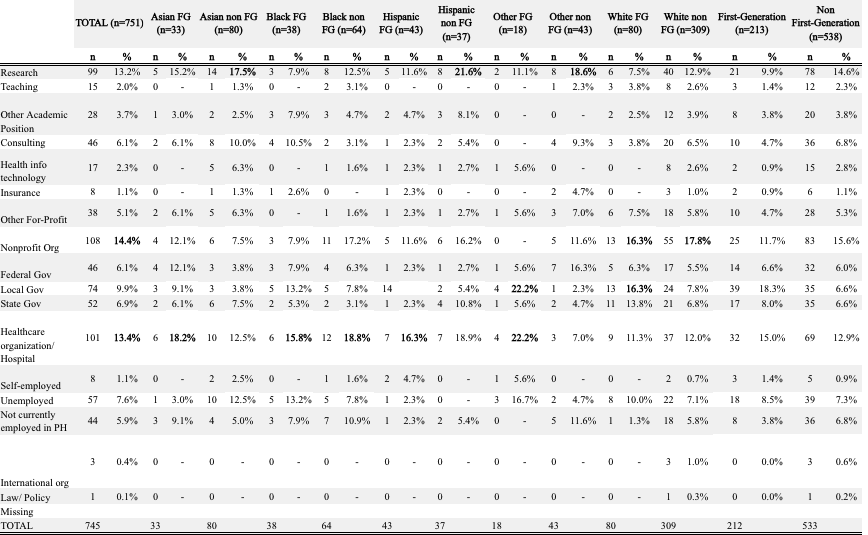


| **Supplemental Table 5. Mean Scores by FG Status (Mann Whitney U-Tests)** | | | | | | |
| --- | --- | --- | --- | --- | --- | --- |
|  | **Total FG(n=213)** | | **Total non-FG (n=538)** | | **TOTAL (n=751)** | |
|  | **Mean** | **SD** | **Mean** | **SD** | **Mean** | **SD** |
| **Job Satisfaction** |  |  |  |  |  |  |
| Job Sat score | 21.7** | 5.04 | 22.8 | 4.5 | 22.5 | 4.7 |
|  |  |  |  |  |  |  |
| **Personal Resources** |  |  |  |  |  |  |
| Self-Efficacy score | 34.5*** | 4.26 | 33.5 | 4.18 | 33.8 | 4.23 |
|  |  |  |  |  |  |  |
| **Social Resources** |  |  |  |  |  |  |
| Social Resource score | 29 | 6.78 | 29.3 | 6.62 | 29.3 | 6.67 |
|  |  |  |  |  |  |  |
| **Economic Resources** |  |  |  |  |  |  |
| Econ Resource score | 9.9** | 3.19 | 9.2 | 3 | 9.4 | 3.01 |
| Comparison of group to non-FG total mean | | | | | | |
| *p<=0.10 |  |  |  |  |  |  |
| **p<=0.05 |  |  |  |  |  |  |
| ***p<=0.001 |  |  |  |  |  |  |

| \| **Supplemental Table 6. Mean Scores by Race/Ethnicity and FG Status (Mann Whitney U-Tests)** \| \| \| \| \| \| \| \| \| \| \| \| \| \| --- \| --- \| --- \| --- \| --- \| --- \| --- \| --- \| --- \| --- \| --- \| --- \| --- \| \|  \| **Asian FG (n=33)** \| \| **Black FG (n=38)** \| \| **Hispanic FG (n=43)** \| \| **Other FG (n= 18)** \| \| **White FG (n=80)** \| \| **Total FG(n=213)** \| \| \|  \| **Mean** \| **SD** \| **Mean** \| **SD** \| **Mean** \| **SD** \| **Mean** \| **SD** \| **Mean** \| **SD** \| **Mean** \| **SD** \| \| **Job Satisfaction** \|  \|  \|  \|  \|  \|  \|  \|  \|  \|  \|  \|  \| \| Job Sat score \| 22.6 \| 5.79 \| 19.6** \| 5.02 \| 21.1 \| 5.2 \| 22.7 \| 4.5 \| 22.4 \| 4.52 \| 21.7 \| 5.04 \| \|  \|  \|  \|  \|  \|  \|  \|  \|  \|  \|  \|  \|  \| \| **Personal Resources** \|  \|  \|  \|  \|  \|  \|  \|  \|  \|  \|  \|  \| \| Self-Efficacy score \| 34.5 \| 5.11 \| 34.6 \| 4.32 \| 34.7 \| 3.63 \| 36.6** \| 4.27 \| 33.9 \| 4.1 \| 34.5 \| 4.26 \| \|  \|  \|  \|  \|  \|  \|  \|  \|  \|  \|  \|  \|  \| \| **Social Resources** \|  \|  \|  \|  \|  \|  \|  \|  \|  \|  \|  \|  \| \| Social Resource score \| 30.9** \| 8.06 \| 30** \| 6.69 \| 29.9* \| 7.03 \| 29.1 \| 7.78 \| 27.3 \| 5.63 \| 29 \| 6.78 \| \|  \|  \|  \|  \|  \|  \|  \|  \|  \|  \|  \|  \|  \| \| **Economic Resources** \|  \|  \|  \|  \|  \|  \|  \|  \|  \|  \|  \|  \| \| Econ Resource score \| 9.3 \| 2.88 \| 10.6* \| 2.41 \| 10.6** \| 2.72 \| 10.9* \| 2.69 \| 9.35 \| 3.44 \| 9.9 \| 3.19 \| \| Comparison of group to FG White mean \| \| \| \| \| \| \| \| \| \| \| \| \| \| *p<=0.10 \|  \|  \|  \|  \|  \|  \|  \|  \|  \|  \|  \|  \| \| **p<=0.05 \|  \|  \|  \|  \|  \|  \|  \|  \|  \|  \|  \|  \| \| ***p<=0.001 \|  \|  \|  \|  \|  \|  \|  \|  \|  \|  \|  \|  \|   **Supplemental Table 7. Mean Scores by Race/Ethnicity and Non-FG Status (Mann Whitney U-Tests)** | | | | | | | | | | | | |
| --- | --- | --- | --- | --- | --- | --- | --- | --- | --- | --- | --- | --- | --- | --- | --- | --- | --- | --- | --- | --- | --- | --- | --- | --- | --- | --- | --- | --- | --- | --- | --- | --- | --- | --- | --- | --- | --- | --- | --- | --- | --- | --- | --- | --- | --- | --- | --- | --- | --- | --- | --- | --- | --- | --- | --- | --- | --- | --- | --- | --- | --- | --- | --- | --- | --- | --- | --- | --- | --- | --- | --- | --- | --- | --- | --- | --- | --- | --- | --- | --- | --- | --- | --- | --- | --- | --- | --- | --- | --- | --- | --- | --- | --- | --- | --- | --- | --- | --- | --- | --- | --- | --- | --- | --- | --- | --- | --- | --- | --- | --- | --- | --- | --- | --- | --- | --- | --- | --- | --- | --- | --- | --- | --- | --- | --- | --- | --- | --- | --- | --- | --- | --- | --- | --- | --- | --- | --- | --- | --- | --- | --- | --- | --- | --- | --- | --- | --- | --- | --- | --- | --- | --- | --- | --- | --- | --- | --- | --- | --- | --- | --- | --- | --- | --- | --- | --- | --- | --- | --- | --- | --- | --- | --- | --- | --- | --- | --- | --- | --- | --- | --- | --- | --- | --- | --- | --- | --- | --- | --- | --- | --- | --- | --- | --- | --- | --- | --- | --- | --- | --- | --- | --- | --- | --- | --- | --- | --- | --- | --- | --- | --- | --- | --- | --- | --- | --- | --- | --- | --- | --- | --- | --- | --- | --- | --- | --- | --- | --- | --- | --- | --- | --- | --- | --- | --- | --- | --- | --- | --- | --- | --- | --- | --- | --- | --- | --- |
|  | **Asian non-FG (n=80)** | | **Black non-FG (n=64)** | | **Hispanic non-FG (n=37)** | | **Other non-FG (n= 43)** | | **White non-FG (n=309)** | | **Total non-FG (n=538)** | |
|  | **Mean** | **SD** | **Mean** | **SD** | **Mean** | **SD** | **Mean** | **SD** | **Mean** | **SD** | **Mean** | **SD** |
| **Job Satisfaction** |  |  |  |  |  |  |  |  |  |  |  |  |
| Job Sat score | 24.2** | 3.67 | 21.2** | 5.43 | 21.9 | 5.08 | 21.5** | 4.33 | 23 | 4.31 | 22.8 | 4.5 |
|  |  |  |  |  |  |  |  |  |  |  |  |  |
| **Personal Resources** |  |  |  |  |  |  |  |  |  |  |  |  |
| Self-Efficacy score | 33.6 | 3.4 | 35** | 3.69 | 33.9 | 3.9 | 31.7* | 5.2 | 33.3 | 4.22 | 33.5 | 4.18 |
|  |  |  |  |  |  |  |  |  |  |  |  |  |
| **Social Resources** |  |  |  |  |  |  |  |  |  |  |  |  |
| Social Resource score | 32*** | 6.81 | 28.9 | 6.21 | 31.3* | 8.26 | 28.2 | 6.18 | 28.7 | 6.31 | 29.3 | 6.62 |
|  |  |  |  |  |  |  |  |  |  |  |  |  |
| **Economic Resources** |  |  |  |  |  |  |  |  |  |  |  |  |
| Econ Resource score | 9 | 3.09 | 9.9** | 2.69 | 10** | 2.54 | 9.5 | 3.14 | 8.9 | 3 | 9.2 | 3 |
| Comparison of group to non-FG White mean | | | | | | | | | | | | |
| *p<=0.10 |  |  |  |  |  |  |  |  |  |  |  |  |
| **p<=0.05 |  |  |  |  |  |  |  |  |  |  |  |  |
| ***p<=0.001 |  |  |  |  |  |  |  |  |  |  |  |  |

**Supplemental Table 8. Impact of Resources on Job Satisfaction by Racial/Ethnic Groups (weighted, adjusted models with interaction)**

|  | **Model 1: Personal Resources** | | | | | | | | | |
| --- | --- | --- | --- | --- | --- | --- | --- | --- | --- | --- |
|  | Asian (n=113) | | Black (n=102) | | Hispanic (n=80) | | White (n=389) | | Total (n=751) | |
|  | **β** | 95% CI | **β** | 95% CI | **β** | 95% CI | **β** | 95% CI | **β** | 95% CI |
| Self-efficacy | 0.44** | 0.18, 0.70 | 0.32 | -0.07, 0.71 | 0.40* | 0.003, 0.79 | 0.17 | -0.06, 0.39 | 0.32** | 0.13, 0.52 |
| First-Generation Status | 2.80 | -13.53, 19.14 | 16.59. | -2.10, 35.27 | 11.71 | -10.05, 33.47 | -7.73 | -20.1, 4.63 | 02.95 | -7.88, 13.78 |
| Interaction | -0.10 | -0.57, 0.36 | -0.57. | -1.14, 0.01 | -0.35 | -0.97, 0.28 | 0.21 | -0.14, 0.57 | -0.11 | -0.41, 0.20 |
|  | **Model 2: Social Resources** | | | | | | | | | |
|  | Asian | | Black | | Hispanic | | White | | Total | |
|  | **β** | 95% CI | **β** | 95% CI | **β** | 95% CI | **β** | 95% CI | **β** | 95% CI |
| Social resource measure | 0.17** | 0.06, 0.29 | 0.36*** | 0.12, 0.54 | 0.31*** | 0.17, 0.45 | 0.12* | 0.01, 0.23 | 0.22*** | 0.12, 0.32 |
| First-Generation Status | -3.70 | -11.87, 4.47 | -10.02. | -20.89, -1.01 | -1.48 | -10.42, 7.47 | -8.04** | -15.25, -0.82 | -4.00. | -8.69, 0.69 |
| Interaction | 0.07 | -0.15, 0.29 | 0.29 | -0.04, 0.65 | 0.003 | -0.26, 0.32 | 0.28* | 0.06, 0.50 | 0.12. | -0.02, 0.25 |
|  | **Model 3: Economic Resources** | | | | | | | | | |
|  | Asian | | Black | | Hispanic | | White | | Total | |
|  | **β** | 95% CI | **β** | 95% CI | **β** | 95% CI | **β** | 95% CI | **β** | 95% CI |
| Economic Resources | 0.42** | 0.16, 0.69 | -0.19 | -0.72, 0.33 | 0.48 | -0.26, 1.22 | -0.09 | -0.34, 0.17 | -0.1 | -0.34, 0.15 |
| First-Generation Status | 1.66 | -4.35, 7.67 | -16.07** | -25.82, -6.33 | 7.18 | -3.51, 17.87 | -7.96*** | -11.78, -4.14 | -5.56** | -9.25,  -1.88 |
| Interaction | -0.33 | -0.93, 0.26 | 1.33** | 0.42, 2.24 | -0.72 | -1.71, 0.27 | 0.87*** | 0.47, 1.28 | 0.54** | 0.16, 0.92 |
| .p<0.10 |  |  |  |  |  |  |  |  |  |  |
| *p<0.05 |  |  |  |  |  |  |  |  |  |  |
| **p<0.01 |  |  |  |  |  |  |  |  |  |  |
| ***p<0.001 |  |  |  |  |  |  |  |  |  |  |
| Covariates for adjusted model include age, gender, marital status, age, degree type, financial, aid, first-generation status  CI, confidence interval | | | | | | | | | | |

## **Supplemental Material. Copy of the Public Health Graduates Survey from SUNY Downstate’s Lambert Lab**

Q4 What is the highest graduate degree or certificate in public health that you have earned? If you have two equivalent degrees (i.e., MPH and MS), please select the most recent degree awarded.

This degree will be referred to as your "highest public health graduate degree/certificate" hereafter.

- MPH (1)
- MS (2)
- DrPH (3)
- PhD (4)
- Advanced Certificate in Public Health (6)
- Other (5) __________________________________________________

Q5 What is the specialty field of your highest public health graduate degree/certificate?

- Allied Health (1)
- Biomedical Sciences (2)
- Biostatistics (3)
- Environmental Sciences (4)
- Epidemiology (5)
- General Public Health (6)
- Global Health (7)
- Health Disparities (8)
- Health Education/behavioral science (9)
- Health Informatics (10)
- Health policy and management (11)
- Maternal and child health (12)
- Nutrition (13)
- Public Health Practice (14)
- Other (15) __________________________________________________

Q6 What year did you earn your highest public health graduate degree/certificate?

________________________________________________________________

Q7 What is the name of the institution that you received your highest public health graduate degree/certificate from?

________________________________________________________________

Q8 Do you have other degrees outside of public health? (e.g., MD, JD, MS in field outside of public health, etc.)

- Yes (please describe your degree(s)): (7) __________________________________________________
- No (1)

Q9 Which of the following financial assistance did you receive to fund any of your public health degrees? Check all that apply.

- Merit-based scholarships (4)
- Financial aid (e.g., student loans, grants, etc.) (5)
- None (7)

Q10 Which sector best fits your **first job in public health (defined below) after completion of your highest public health graduate degree/certificate**? If you have recently graduated and accepted a job, but not started yet, please answer for that job's sector. 

 Public health is a diverse field and employs professionals in numerous job functions within government agencies, research institutes, universities, hospitals, nonprofit organizations, and corporations. Government agencies, including federal government, local health departments (LHDs), and state, tribal, and territorial health departments (SHDs) play unique roles in the public health system, including disease surveillance, reporting, screening, treatment and counseling; laboratory testing; vaccine inventory and distribution; food safety; behavioral health; regulatory inspection and licensing; emergency response; maternal/child health and newborn screening; HIV and substance use disorder prevention; and nutrition.

- Research (1)
- Teaching (2)
- Other Academic position (3)
- Consulting (4)
- Health information technology (5)
- Insurance (6)
- Other For-profit (7)
- Nonprofit (8)
- Federal (9)
- Local (10)
- State (11)
- Healthcare organization/Hospital (12)
- Self-employed (13)
- Other employment sector (please describe): (14) __________________________________________________
- Was not employed after graduation (15)

Q11 What was your position at this job?

________________________________________________________________

Q12 Which employment sector best fits your **current job in public health**? If this is the same job as your first public health job after completion of degree/certificate, please select "same job as first job after graduation" below.

- Research (1)
- Teaching (2)
- Other Academic position (3)
- Consulting (4)
- Health information technology (5)
- Insurance (6)
- Other For-profit (7)
- Nonprofit (8)
- Federal (9)
- Local (10)
- State (11)
- Healthcare organization/Hospital (12)
- Self-employed (13)
- Other employment sector (please describe): (14) __________________________________________________
- Not currently employed anywhere (15)
- Not currently employed in a public health job (19)
- Same job as first job after graduation (18)

Q13 What is your position at your current job?

________________________________________________________________

Q15 Please indicate how strongly you agree or agree with the following statements:

|  | **Strongly Agree (1)** | **Agree (2)** | **Neither agree nor disagree (3)** | **Disagree (4)** | **Strongly Disagree (5)** |
| --- | --- | --- | --- | --- | --- |
| I am happy with **my current employment sector.** (1) |  |  |  |  |  |
| I am happy with the **amount of opportunities to advance in my current position.** (2) |  |  |  |  |  |
| I am happy with **my current salary.** (3) |  |  |  |  |  |
| I am happy with the **level of responsibility in my position.** (4) |  |  |  |  |  |
| I enjoy the **people that I work with.** (6) |  |  |  |  |  |
| I hope to stay in **my current employment sector** long term. (5) |  |  |  |  |  |

Q16 Please indicate how strongly you disagree or agree with the following statements about the choice of your employment sector (i.e., Academia, For-Profit, Non-Profit, Government, Healthcare organization/Hospital, Self-employed):

|  | **Strongly Agree (1)** | **Agree (2)** | **Neither agree nor disagree (3)** | **Disagree (4)** | **Strongly Disagree (5)** |
| --- | --- | --- | --- | --- | --- |
| The choice of my current employment sector was highly influenced by **job availability**. |  |  |  |  |  |
| The choice of my current employment sector was highly influenced by  **my level of student debt**. |  |  |  |  |  |
| The choice of my current employment sector was highly influenced by the **geographical location of where I wanted to work.** |  |  |  |  |  |
| The choice of my current employment sector was highly influenced by **my need (or desire) to earn more money**. |  |  |  |  |  |
| The choice of my current employment sector was highly influenced by the **financial prospects of the career sector**. |  |  |  |  |  |
| The choice of my current employment sector was highly influenced by the sector's  **positive working conditions (hours, people, work environment).** |  |  |  |  |  |
| **Guidance from mentors** has highly influenced my current employment sector. |  |  |  |  |  |
| The choice of my current employment sector was highly influenced by **interests in a specific field of public health**. |  |  |  |  |  |

Q17 Please indicate how strongly you disagree or agree with the following statements about the choice of your employment sector (i.e., Academia, For-Profit, Non-Profit, Government, Healthcare organization/Hospital, Self-employed):

|  | **Strongly Agree (1)** | **Agree (2)** | **Neither agree nor disagree (3)** | **Disagree (4)** | **Strongly Disagree (5)** |
| --- | --- | --- | --- | --- | --- |
| The choice of my current employment sector was highly influenced by **responsibility to my family (significant other/spouse, children, and/or other dependents)** |  |  |  |  |  |
| The choice of my current employment sector was highly influenced by my experiences throughout my **graduate education (coursework, internships, and other training)** |  |  |  |  |  |
| The choice of my current employment sector was highly influenced by **formal career counseling in my graduate education.** |  |  |  |  |  |
| The choice of my current employment sector was highly influenced by **a commitment to a personal/collective mission or purpose.** |  |  |  |  |  |
| The choice of my current employment sector was highly influenced by **the prestige that comes with the position.** |  |  |  |  |  |
| The choice of my current employment sector was highly influenced by **my gender representation in the field.** |  |  |  |  |  |
| The choice of my current employment sector was highly influenced by **my racial/ethnic representation in the field.** |  |  |  |  |  |
| The choice of my current employment sector was highly influenced by **my immigration status or citizenship.** |  |  |  |  |  |
| The choice of my current employment sector was highly influenced by **my cultural values (i.e., religious or familial values).** |  |  |  |  |  |
| The choice of my current employment sector was highly influenced by  **my family's occupational background (e.g., I have a family member who is in academia/government/a healthcare organization or hospital, etc.)** |  |  |  |  |  |

Q19 Please indicate how strongly you disagree or agree with the following statements:

|  | **Strongly agree (1)** | **Agree (2)** | **Neither agree nor disagree (3)** | **Disagree (4)** | **Strongly disagree (5)** |
| --- | --- | --- | --- | --- | --- |
| I will be able to achieve most of the goals that I have set for myself. (1) |  |  |  |  |  |
| When facing difficult tasks, I am certain that I will accomplish them. (2) |  |  |  |  |  |
| In general, I think that I can obtain outcomes that are important to me. (3) |  |  |  |  |  |
| I believe I can succeed at most any endeavor to which I set my mind. (4) |  |  |  |  |  |
| I will be able to successfully overcome many challenges. (5) |  |  |  |  |  |
| I am confident that I can perform effectively on many different tasks. (6) |  |  |  |  |  |
| Compared to other people, I can do most tasks very well. (7) |  |  |  |  |  |
| Even when things are tough, I can perform quite well. (8) |  |  |  |  |  |

Q20 We want to collect input from a diverse array of people living in the US, including people who are not citizens or permanent residents. Are you a United States citizen or permanent resident of the U.S.?

- Yes (1)
- No (2)
- Choose not to answer (3)

Q21 Ethnicity:

- Hispanic or Latinx (1)
- Not Hispanic or Latinx (2)

Q22 Race (check all that apply):

- Asian American (e.g., Chinese, Filipino, Asian Indian, Vietnamese, Korean, Japanese) (1)
- Black (e.g., African American, Jamaican, Haitian, Nigerian, Ethiopian, Somalian) (2)
- Indigenous, First Nations, American Indian, or Alaska Native (e.g., Aboriginal, Navajo Nation) (3)
- Middle Eastern or North African (7)
- Native Hawaiian or other Pacific Islander (e.g. Samoan, Chamorro, Tongan, Fijian) (4)
- White or European American (5)
- Other, please specify (6) __________________________________________________

Q23 What is the gender by which you most identify?

- Male (4)
- Female (5)
- Self-describe (e.g., Trans Male/Trans Man, Trans Female/Trans Woman, Genderqueer/Gender Nonconforming, Other, etc.) (7) __________________________________________________

Q24 Are you the first generation of your family to complete a college degree? First generation is defined as someone whose parents did not complete a college degree.

- Yes (1)
- No (2)

Q25 Have you received or been eligible for a low-income (need-based) grant or loan as a student (e.g. Pell grant, New York State Tuition Assistance Program) or any other public assistance (e.g., SNAP, free or reduced school lunch, etc.)?

- Yes (1)
- No (2)
- Unsure (3)

Q26 Marital Status:

- Currently Married (1)
- Widowed (2)
- Divorced (3)
- Separated (10)
- Never Married (11)
